# The Role of Observation Scales, Trait Correlations, and Competitive Regimes in Community Assembly Patterns

**DOI:** 10.1002/ece3.71272

**Published:** 2025-04-24

**Authors:** Matthias Rohr, Wilfried Thuiller, Loïc Chalmandrier, Tamara Münkemüller

**Affiliations:** ^1^ CNRS, LECA Université Grenoble Alpes, Université Savoie Mont Blanc Grenoble France; ^2^ CNRS, Inria Université Grenoble Alpes Grenoble France

**Keywords:** environmental filter, fitness difference, functional diversity, limiting similarity, trait patterns

## Abstract

Inferring assembly processes from empirical community diversity patterns has long been a central goal in Ecology. Many studies rely on the “filtering framework,” which characterizes community assembly as a sequence of abiotic and competitive interactions. The framework's success stems from its theoretical foundation, linking environmental filtering to niche theory and competitive interactions to coexistence theory. Empirical studies have provided substantial evidence for environmental filtering across diverse ecosystems. However, despite the ubiquity of competitive interactions, empirical applications of the filtering framework have rarely detected significant competition effects. Consequently, the framework has been criticized as overly simplistic. We argue that this imbalance arises from three conceptual challenges. First, empirical studies often use traits without clearly distinguishing between those that capture species' responses to the environment versus those that mediate competition, nor do they consider how these trait sets may co‐vary. Second, the framework overlooks that environmental filtering and competition can produce similar trait patterns. Third, the spatial scale at which communities are observed strongly impacts the resulting trait patterns. Here, we explore these three challenges and test how trait patterns vary depending on different assembly processes, trait correlations, and spatial scales. Using a theoretical simulation model, we demonstrate that trait patterns resulting from environmental filtering and competition exhibit different sensitivities to variations in trait correlation structure and observation scales. We then identify the conditions under which distinct assembly processes can be inferred from observed trait patterns, given the correlation and relevance of traits and the inherent constraints of the observational scales.

## Graphical abstract

Ecologists have long used the ecological filtering framework to disentangle community assembly processes from trait distribution data. To derive clearer expectations from the filtering framework, we developed a spatially explicit trait‐based community model that considers the scale at which communities are observed, fine‐scale environmental heterogeneity, both symmetric and asymmetric competitions, and multiple traits. We found that trait correlations limit the identifiability of community assembly processes using trait‐based methods. This discovery has serious consequences for empirical assessments since ecologists usually consider various “soft” traits that show strong dependencies and trade‐offs.

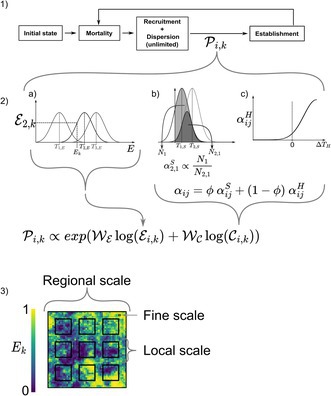

## 1 Introduction

A fundamental challenge in ecology is predicting how environmental spatial variations influence the composition, structure, and functioning of ecological communities. Environmental variations directly impact species through physiological constraints, mortality, and fecundity while also affecting them indirectly via competitive interactions (Alexander et al. 2015). In the face of ongoing global environmental changes, understanding how abiotic and biotic environments drive community assembly is crucial for predicting long‐term ecosystem functioning and nature's contribution to people.

To address this challenge, ecologists have long employed the ecological filtering framework to infer community assembly processes from empirical data (Diamond 1975; Keddy 1992). This framework posits that local communities result from a sequence of environmental filters and competitive interactions. Initially, environmental filters select species from the regional pool based on their ability to tolerate local environmental conditions (i.e., their fundamental niche). Competitive interactions subsequently determine which species can coexist in a local community (Chesson 2000). Early applications of this framework contrasted two mere assumptions: environmental filtering leads to communities of species with similar environmental requirements (i.e., species with the same niche), while the competitive interactions reduce species niche similarity in the communities due to competitive exclusion (i.e., limiting similarity, Mouillot et al. 2007). Consequently, dominant assembly processes were inferred by examining similarity patterns in observed community structures (Münkemüller et al. 2020).

Functional traits, which are morphological, physiological, or phenological features affecting species performance (Violle et al. 2007), serve as key proxies for niche similarity (Thuiller et al. 2004). Since environmental filtering reduces the possible range of viable strategies, it typically leads to trait similarity (Cornwell et al. 2006). In contrast, limiting similarity theory (Macarthur and Levins 1967) suggests that competition fosters coexistence of species with dissimilar traits (Cornwell et al. 2006), named trait dissimilarity.

Despite its overall simplicity, the ecological filtering framework's success lies in its theoretical foundation, linking environmental filtering to niche theory and competitive interactions to coexistence theory (Grinnell 1917; Hutchinson 1957; Chesson 2000). As such, the filtering framework mixes these two theoretical corpuses. It has provided evidence of environmental filtering in a wide range of environments (Cornwell et al. 2006; Kraft et al. 2008; Cornwell and Ackerly 2009; Chalmandrier et al. 2017; de Bañares‐Dios et al. 2020). The framework's flexibility also allows for refinements, such as multiple scale analysis (Chalmandrier et al. 2013, 2017; Götzenberger et al. 2016), and to account for single or multiple traits to measure species similarity (Spasojevic and Suding 2012).

However, few studies have found evidence for limiting similarity processes (Götzenberger et al. 2012), and those have primarily focused on low‐diversity systems such as salt marshes (Wilson and Stubbs 2012) or sand dunes (Stubbs and Wilson 2004). In contrast, many empirical studies highlight the overwhelming role of environmental filtering (Münkemüller et al. 2020). One possible explanation is that limiting similarity may be less influential in species‐rich communities (de Bañares‐Dios et al. 2020). However, we argue that the filtering framework, as commonly applied, oversimplifies natural systems by neglecting fine‐scale environmental heterogeneity (Willis et al. 2010), alternative competition regimes such as hierarchical competition (Mayfield and Levine 2010), and trait correlation complexities (Kunstler et al. 2012).

More specifically, the framework assumes homogeneous local environments, yet fine‐scale heterogeneity can counteract broader‐scale environmental filtering effects. For instance, microhabitat variation in soil conditions over a few centimeters may drive subtle trait differences within a plot, potentially leading to apparent trait dissimilarity misinterpreted as competition (Bello et al. 2013; Willis et al. 2010). In addition, limiting similarity through symmetric competition (Macarthur and Levins 1967) is not the unique competitive regime that happens in natural communities. For example, certain functional traits may offer a higher competitive ability (i.e., fitness difference in Chesson 2000 theory) to some species, for example, plant height for light competition (Kunstler et al. 2012). Competitive ability differences, often hierarchic competition in the filtering framework literature (Münkemüller and Gallien 2015; Münkemüller et al. 2020; Bektaş et al. 2023), can lead to species in communities having similar traits (Mayfield and Levine 2010). In other words, hierarchic competition can lead to similar observed patterns than expected under large‐scale environmental filtering (high species, trait similarity), while fine‐scale environmental heterogeneity can lead to similar patterns than symmetric competition (low species similarity, trait dissimilarity).

Interestingly, these mixed expectations reflect a latent vagueness around how to define species ecological similarity through traits and how to infer the assembly processes at play from trait patterns. On one hand, in most theoretical work on community assembly processes, a single trait is assumed to quantify both species' responses to environmental gradients but also the competitive interactions between species, commonly defined as a function of the overlap between environmental traits (e.g., Münkemüller and Gallien (2015), Munoz et al. (2018), and Chalmandrier et al. (2022)). On the other hand, when analyzing empirical trait patterns, ecologists do not expect that a single trait captures both environmental and competitive interactions (Bello et al. 2013; Rosbakh et al. 2022). Instead, they commonly employ several traits that loosely include information on how the species responds to the environment but also affect other species (Lavorel and Garnier 2002; Mouillot et al. 2021). How these traits covary with each other depends on trade‐offs in phenotype construction (Pigliucci 2003) and will likely influence the outcome of trait pattern analyses. Yet, we still lack clear expectations on the effects of community assembly processes on trait patterns and how they will be influenced by the correlation structure among traits.

To address these issues, we extend the filtering framework to incorporate fine‐scale environmental heterogeneity, symmetric and hierarchic competition, and multiple traits and their correlation to reflect both environmental responses and competitive interactions. Through simulations, we establish theoretical expectations for community trait patterns and evaluate whether observed trait patterns can reliably differentiate between assembly processes. Crucially, we assess how observational scale (i.e., the spatial grain, at which communities are observed by ecologists) influences inference accuracy, recognizing that defining community boundaries is itself non‐trivial. Specifically, we ask: (i) Do fine‐scale environmental filters and symmetric competition lead to distinct trait patterns at different spatial scales, and does the correlation structure between traits affect these patterns? (ii) Do symmetric and hierarchic competition lead to distinct trait patterns at different scales when they act in concert with environmental filtering? (iii) Under what conditions can distinct assembly processes be reliably inferred from observed trait patterns?

## 2 Theoretical Model and Methods

### 2.1 Model Description

#### 2.1.1 Global Overview

**FIGURE 1:**
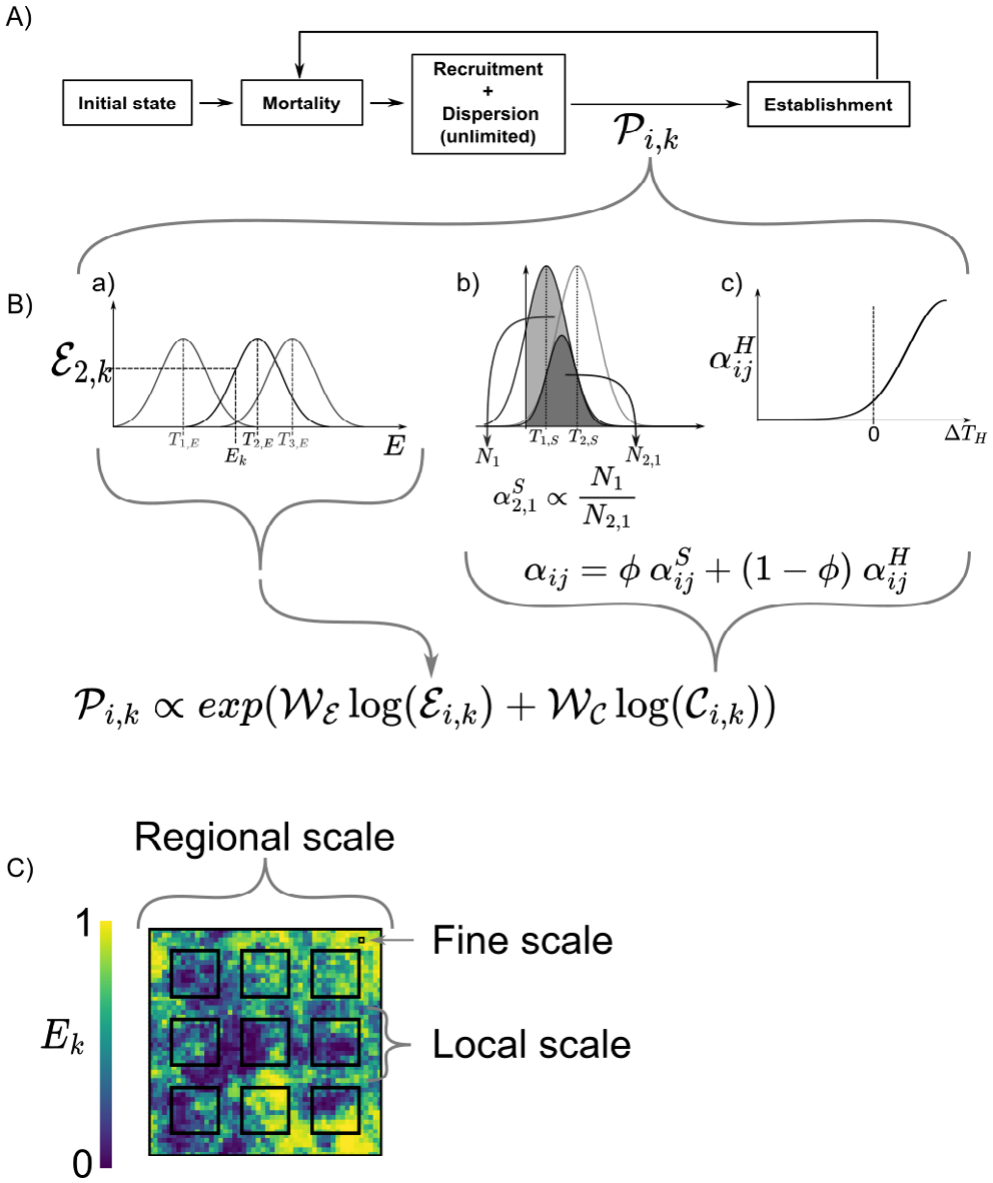
Schematiic representation of the simulated processes in the model. (A) Flowchart describing the scheduling of the simulation. The colonization probability $P_{i,k}$ for a given species i in a cell *k* depends on both environmental filter and competitive interactions. (B) Schematic overview of the various assembly processes. (a) The environmental filter is defined by species' performance relative to the environmental value ($E_{k}$) of the cell and its optimum trait ($T_{i,E}$). (b) Symmetric competition is determined by the niche overlap between species. (c) Hierarchic competition is modeled as hierarchic traits difference between species $\Delta T_{H}=T_{i,H}-T_{j,H}$. (C) Distribution of the environmental conditions ($E_{k}$) and the three different observation scales at which the simulated communities are sampled.

We developed a spatially explicit, individual‐based model to investigate how environmental filtering, symmetric competition, and hierarchical competition jointly influence community assembly. Our primary objective was to derive theoretical expectations about emergent trait patterns in response to (i) fine‐scale environmental heterogeneity within observed communities, (ii) the interplay between symmetric and hierarchical competition, and (iii) correlations among traits associated with environmental responses and competitive interactions. Although originally conceived for plants, the model can be applied to any non‐trophic system. Below, we outline the main components of the model, including its general workflow, the community assembly process, and the spatial structure of the environmental factors, along with the scales of observation (Figure 1).

The model is implemented as a cellular automaton, where each cell harbors a local community of individuals with a maximum total abundance of *K*. At the start of each simulation, we generate a regional species pool of size *S*. Each species is characterized by three functional traits that determine (i) environmental preference, (ii) symmetric competitive effects, and (iii) hierarchical competitive advantage relative to other species. During an initialization phase, individuals from the species pool are placed randomly on the landscape until each cell reaches its carrying capacity. The model then proceeds through iterative “reassembly” steps, beginning with stochastic mortality (with a fixed probability for all individuals), followed by recruitment determined by environmental filtering and competitive effects. After 3000 time steps (sufficient for local trait diversity to stabilize), communities are sampled at multiple spatial scales (one cell, a 10 *×* 10 cell site, or the entire landscape) to quantify emergent trait patterns with commonly used measures in empirical diversity analyses (see Section 2.3).

More specifically, we consider a species pool of _*S*_ species. Each species has three functional traits: *T*_Env_, *T*_Sym_, and *T*_Hier_.

- *T*_Env_, the “*environmental trait*,” determines the species' response to the environment;
- *T*_Sym_, the “*symmetric trait*,” is an effect trait governing the symmetric competition a species has with the others;
- *T*_Hier_, the “*hierarchic trait*,” defines a species' dominance or hierarchical advantage over others (Figure 1).

All traits are assigned at the species level without intraspecific variation and are drawn from a Gaussian copula to allow for specified correlations among the traits while preserving a uniform [0, 1] marginal distribution.

We modeled the landscape on a toroidal *n × n* grid, in which each cell has an associated environmental value *E*_*k*_. To investigate the impact of fine‐scale environmental heterogeneity on observed trait patterns, we generated auto‐correlated environmental landscapes following Viana and Chase (2019), with the “range” parameter (Table 1) specifying the spatial autocorrelation. The environmental values (*E*_*k*_) were drawn to remain uniformly distributed across the grid, preventing bias in trait patterns due to skewed environmental distributions. A species' environmental trait, *T*_Env_, determines its optimal position along the environmental axis, and the strength of the environmental filtering depends on the match between *T*_Env_ and *E*_*k*_.

**TABLE 1:** Parameters and values of the simulation experiments.

Name	Description	Values
*S*	Number of species in the regional species pool	100
*n*	Size of the landscape grid	50
*K*	Carrying capacity of the cells	10
μ	Mortality rate	0.2
Range	Autocorrelation of the Gaussian field that defines the landscape	5.0
$W_{E}$	Strength of the environmental filtering	1
$W_{C}$	Strength of competition	10
*F*	Fecundity rate	0.1
ω	Environmental niche breadth	0.1
$\sigma_{S}$	Symmetric competitive niche breath	0.1
$\sigma_{H}$	Hierarchic competitive niche breath	0.2
ϕ	Relative importance of symmetric versus hierarchic competition in $\alpha_{i,j}$	0–0.5—1
*c*	Correlation coefficient between *T*_Env_, *T*_Sym_, and *T*_Hier_	0–0.5—0.8—1

Community assembly begins with the aforementioned random initialization, where each cell is filled to its carrying capacity *K* with individuals drawn from the species pool according to a multinomial distribution with equal probability for all species (1*/S*). The model then iterates through mortality and recruitments.

Mortality: Each individual dies with a fixed, species‐independent probability μ (see Table 1, Figure 1A).

Recruitment: Vacated “slots” are refilled following a weighted lottery that incorporates both environmental filtering and competitive interactions (Figure 1B, Equation (1)). The weight ($P_{i,k}$) for species *i* in cell *k* depends on (i) environmental filtering, $E_{i,k}$, (ii) competition, $\left(C_{i,k}\right)$, and (iii) the regional abundance *A*_*i*_ (multiplied by a fecundity factor *F*):

(1) $$P_{i,k}=\exp\left(W_{E}\;\log\left(E_{i,k}\right)+W_{C}\;\log\left(C_{i,k}\right)\right)FA_{i}$$

where $W_{E}$ and $W_{C}$ are the relative strength of environmental filtering and competition, respectively. All $P_{i,k}$ are then normalized among species within each cell to yield probabilities of colonization.

#### 2.1.2 Details of the Processes

The environmental filter $E_{i,k}$ measures how well species *i* is suited to the environment of cell *k*. It is given by a Gaussian function centered at $T_{\mathrm{Env}}^{i}$ with a common niche breadth $\omega_{i}$ for all species (Figure 1B.a, Equation (2)):

(2) $$E_{i,k}=\exp\left(-\frac{{\left(T_{\mathrm{Env}}^{i}-E_{k}\right)}^{2}}{2\;\omega_{i}^{2}}\right)$$

The competition filter $C_{i,k}$ is defined as the sum of all species pairwise competitive coefficients ($\alpha_{i,j}$) weighted by the abundance of each species *j* in cell *k*, *ab*_*j,k*_ (Equation 3):

(3) $$C_{i,k}=\sum_{j}\alpha_{i,j}\;ab_{j,k}$$

The pairwise coefficients $\alpha_{i,j}$ combine symmetric and hierarchical competition (Equation 4):

(4) $$\alpha_{i,j}=\phi \;\alpha_{i,j}^{S}+\left(1-\phi \right)\;\alpha_{i,j}^{H}$$

where ϕ *∈* [0, 1] controls the relative importance of symmetric ($\alpha_{i,j}^{S}$) and hierarchic ($\alpha_{i,j}^{H}$) competitive coefficients (Chalmandrier et al. 2022). ϕ allows for various assembly scenarios: exclusively symmetric competition ϕ = 1, exclusively hierarchic competition ϕ = 0, or a mixed regime ϕ = 0.5. Competitive coefficients are derived from species' competitive traits (*T*_Sym_ and *T*_Hier_).

- Symmetric competition ($\alpha_{i,j}^{S}$): We assume that symmetric competition depends on species' similarity along a “resource axis” *L ∈* [0, 1]. Each species *i* has a Gaussian niche $N_{i}\left(L\right)$ centered at $T_{\mathrm{Sym}}^{i}$ with a common breadth $\sigma_{S}$. We compute $\alpha_{i,j}^{S}$ as the overlap of species *i*'s and *j*'s niches, normalized by the area of *j*'s niche (Equation 5a). This approach balances edge effects by penalizing species at the boundaries (Scheffer and Van Nes 2006):
(5a) $$N_{i}\left(L\right)=\exp\left(-\frac{{\left(T_{\mathrm{Sym}}^{i}-L\right)}^{2}}{2\;\sigma_{s}^{2}}\right)$$
(5b) $$\alpha_{i,j}^{S}=\frac{\int_{0}^{L_{\max}}N_{i}\left(L\right)\;N_{j}\left(L\right)\;\mathrm{dL}}{\int_{0}^{L_{\max}}N_{j}{\left(L\right)}^{2}\;\mathrm{dL}}$$

Hierarchic competition ($\alpha_{i,j}^{H}$): We assume that species with higher values of $T_{\text{Hier}}^{i}$ exert a stronger competitive effect on species with lower values. The hierarchical competition coefficient between species *i* and *j* is given by a Gaussian function centered at $\left(T_{\text{Hier}}^{i}-T_{\text{Hier}}^{j}\right)-1$, with breadth $\sigma_{h}$. Larger $\sigma_{h}$ values reduce this minimal trait difference. Consequently, species with higher hierarchic trait values exert a stronger influence on species with lower trait values.

Intraspecific competition is set to 1 (Equation 6):

(6) $$\alpha_{i,j}^{H}=\left\{\begin{array}{c} \exp\;\left[-\frac{1}{2}{\left(\frac{T_{\text{Hier}}^{i}-T_{\text{Hier}}^{j}}{\sigma_{h}}\right)}^{2}\right]\;\text{if}\;i\neq j\\ 1\;\text{if}\;i\neq j \end{array}\right.$$

Figure 1 illustrates the scheduling of these processes and provides a schematic of how environmental filtering, symmetric competition, and hierarchical competition are calculated.

### 2.2 Simulation Experiments

We conducted three simulation experiments to determine how emergent trait patterns are affected by (i) trait correlations, (ii) the relative importance of symmetric versus hierarchical competition, and (iii) the spatial scale of observation:

- Experiment 1: We examined whether environmental filtering and symmetric competition can be distinguished at different scales when *T*_Env_ and *T*_Sym_ are correlated. Here, we set *ϕ* = 1 (purely symmetric competition) and varied the correlation coefficient *c* among the three traits ($c\in \left\{0,0.5,0.8,\;1\right\}$, Table 1). All three traits (*T*_Env_, *T*_Sym_, and *T*_Hier_) were included, allowing us to explore whether an apparently “neutral” trait (here *T*_Hier_) can generate patterns solely through its correlations with other traits.
- Experiment 2: We tested the role of different competition regimes by varying *ϕ* between 0 and 1 ($\phi \in \left\{0,0.5,1\right\}$), while keeping all traits independent (*c* = 0). This allowed us to assess how purely hierarchical, purely symmetric, or mixed‐competition regimes shape trait patterns.
- Experiment 3: We explored the full parameter space of both ϕ and *c* ($\phi \in \left\{0,0.5,1\right\};c\in \left\{0,\;0.5,0.8,\;1\right\}$) to identify the range of potentially distinguishable trait patterns under environmental filtering, symmetric and hierarchical competitions.

For each parameter combination, we ran 100 replicate simulations to account for stochasticity in species‐pool generation and community assembly. Table 1 lists both the fixed parameters and the parameter values that were systematically varied in our experiments. A sensitivity analysis for fixed parameters is provided in Supporting Information.

### 2.3 Trait Patterns

We evaluated trait patterns at three observation scales (Figure 1C) to capture the interplay between local interactions and environmental heterogeneity:

1. Regional scale: The entire *n × n* grid (analogous to large‐scale surveys)
2. Local scale: Nine sites of 10 *×* 10 cells each (analogous to quadrat surveys)
3. Fine scale: Single cells (analogous to very fine‐scale surveys)

These observation scales reflect the relationship between environmental filtering, competitive interactions, and sampling intensity: “fine scale” corresponds to the level at which individuals directly interact (within cells [e.g., de Bello et al. (2013); Bektaş et al. (2023)]); “local scale” is too coarse for all individuals to actively interact and captures low levels of environmental heterogeneity (de Bello et al. 2013; Gaüzère et al. 2023); and “regional scale” represents largescale environmental heterogeneity (de Bello et al. 2013; Gaüzère et al. 2023). The real‐world relevance of these scales depends on species' zones of influence and the degree of environmental variability in the system.

Because observed trait patterns can be influenced by differing sample sizes across scales, we used a resampling approach to ensure the same total number of individuals per “observation” at each scale (Bektaş et al. 2023). Specifically, for the regional and local scales, we randomly sampled *K* individuals with probabilities proportional to their abundances, creating 80 replicate samples. For the fine scale, we selected 80 cells at random. This procedure yields comparable community matrices (observation × species) at each scale.

To quantify trait diversity, we used Rao's quadratic entropy (Rao 1982) for each trait separately and for all traits combined (multidimensional trait diversity). We then compared observed trait diversity to null expectations using 200 randomizations of the community matrix. For each site, we randomly shuffled the identity of the observed species among all species in the pool generated at the initial states (following the same algorithm as “*richness*” implemented in the R package *Picante*, Kembel et al. 2010). This randomization procedure preserves species richness and abundances constant between observations and randomizations. We computed the standardized effect size (SES) for each observation (Equation 7):

(7) $$\mathrm{SES}=\frac{{\text{Metric}}_{\mathrm{obs}}-\text{mean}\left({\text{Metric}}_{\text{null}}\right)}{\mathrm{sd}\left({\text{Metric}}_{\text{null}}\right)}$$

Negative or positive values of SES values indicate that observed trait diversity is, respectively, lower or higher than expected under a random assembly process.

Overall, this modeling framework allows us to generate systematic expectations about how environmental filtering, symmetric competition, and hierarchical competition interact to shape trait patterns, and how those patterns manifest at different spatial scales. We focus on the significance of trait correlations, the relative contribution of different competitive modes, and the sampling scale's influence on detecting these mechanisms.

## 3 Results

### 3.1 General Overview

The simulations produced diverse communities, with Shannon index values ranging from 29.0 to 97.3 (mean: 68.4 ± 16.3) at the regional scale, across the three simulation experiments.

In a control scenario without trait correlations and environmental heterogeneity (i.e., a homogeneous environmental landscape), the simulated trait diversity patterns aligned with classical expectations from the filtering framework. Specifically, symmetric competition promoted high diversity in the symmetric trait, whereas both hierarchical competition and environmental filtering led to lower‐than‐expected diversity in the hierarchical and environmental traits. These patterns were consistent across all observation scales (see Supporting Information). The following sections focus on results obtained in heterogeneous environments.

Patterns of multidimensional trait diversity largely reflected the combined effects of individual trait patterns. In most cases, multidimensional diversity was lower than expected under random assembly, primarily due to the combined effects of hierarchical competition and environmental filtering, both of which reduced trait diversity. However, under strong symmetric competition, the opposing effects of environmental filtering (which reduces diversity) and competition (which increases diversity) resulted in trait diversity patterns resembling those expected under random assembly (Supporting Information). Overall, multidimensional trait diversity did not reliably distinguish the simultaneous action of multiple assembly processes. For these reasons, the following results focus on single‐trait diversity patterns.

### 3.2 Influence of Trait Correlation and Observation Scale on Community Trait Patterns

**FIGURE 2:**
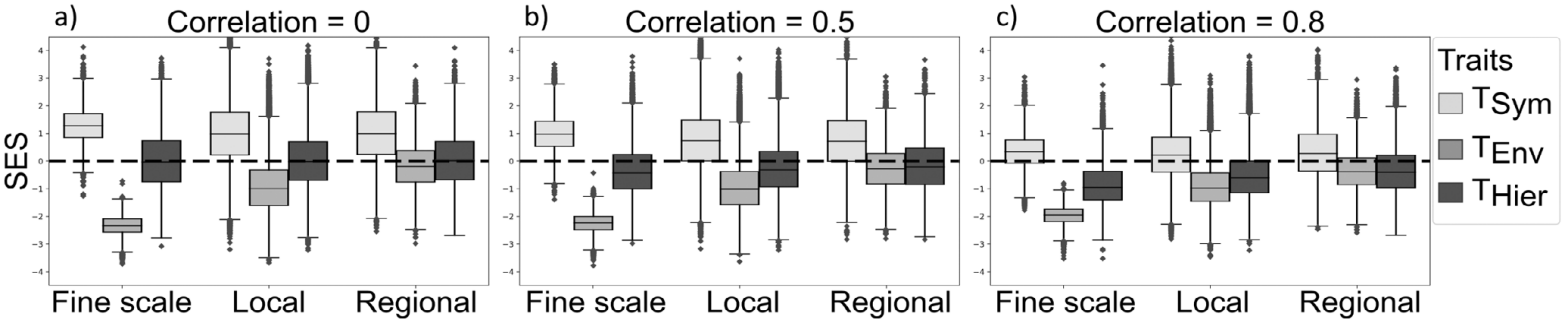
Standardized effect size (SES) of trait diversity for the three traits across different scales of observation: Fine scale (cell's communities), Local scale (10 *×* 10 cell units), and Regional scale (entire landscape). Three different simulation experiments are shown: (a) No trait correlation, (b) Moderate trait correlation (correlation coefficient *c* = 0.5), and (c) High trait correlation (correlation coefficient *c* = 0.8) *T*_Sym_, *T*_Env_, and *T*_Hier_ correspond to traits associated to symmetric competition, environmental filtering, and hierarchic competition, respectively. The simulations considered only environmental filtering and symmetric competition as acting processes (ϕ = 1).

For simulated communities experiencing symmetric competition, when traits were uncorrelated and observed at the fine scale (i.e., no environmental heterogeneity within observed communities), the resulting trait patterns matched theoretical expectations from the filtering framework (Figure 2a). Specifically, diversity of the symmetric trait (*T*_Sym_) was higher than expected under random assembly (mean SES = 1.02, 95% CI: [1.02, 1.03]), that is, indicating trait dissimilarity. Meanwhile, diversity of the environmental trait (*T*_Env_) was lower than expected, indicating trait similarity (mean SES = *−*2.34, 95% CI: [*−*2.35, *−*2.33]). Given the absence of hierarchical competition in these simulations, hierarchical trait diversity (*T*_Hier_) followed a random pattern (mean SES = 0.02, 95% CI: [0.00, 0.04]). These results confirm that the model generates theoretically expected trait patterns in simple scenarios, providing a baseline for interpreting more complex assembly processes.

Where trait correlations were low to moderate (*c* ≤ 0.5, Figure 2b), the symmetric trait (*T*_Sym_) still exhibited higher than expected diversity (mean SES = 0.52, 95% CI: [0.52, 0.53]), a pattern that remains stable across the three observation scales. However, as trait correlations increased, this signal weakened, shifting toward randomness at high correlations (*c* = 0.8, Figure 2c), particularly at the broader observation scales.

In contrast, the environmental trait (*T*_Env_) exhibited diversity patterns that were largely insensitive to trait correlations (Figure 2). Trait diversity was consistently lower than expected, but its detectability varied with observation scale. Specifically, at fine scales, strong trait similarity was evident (mean SES = *−*0.88, 95% CI: [*−*0.89, *−*0.87]), whereas at regional scales, diversity patterns approached those expected under random assembly (mean SES = 0.01, 95% CI: [0.00,0.02]).

### 3.3 Distinguishing Trait Patterns Under Different Competitive Regimes and Observation Scales

**FIGURE 3:**
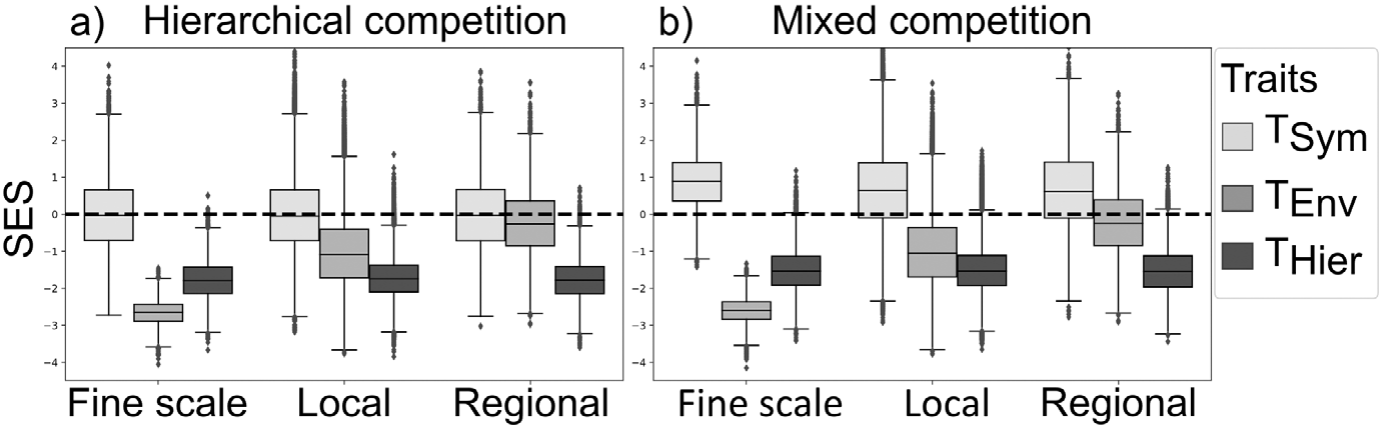
Standardized effect size (SES) of trait diversity for the three functional traits as a function of the observation scale: (a) Hierarchic competition is the only interspecific interaction regime involved in community assembly (ϕ = 0); (b) species interact through both symmetric and hierarchic competition (ϕ = 0.5).

When hierarchical competition was introduced, trait diversity patterns at fine and local observation scales aligned with theoretical expectations from the filtering framework (Figure 3). The environmental (*T*_Env_) and hierarchical (*T*_Hier_) traits consistently displayed lower diversity compared to random assembly, while the diversity of symmetric trait (*T*_Sym_) deviated from random expectation only when symmetric competition was active in the assembly processes (Figure 3b). Notably, at these observation scales, environmental filtering and hierarchical competition produced qualitatively indistinguishable patterns.

Similar to symmetric competition, hierarchical competition produced consistent trait diversity patterns across observation scales. In contrast, the environmental trait showed a clear‐scale dependent pattern, transitioning from lower than expected trait diversity at fine scale to randomlike patterns at regional scale. This difference in scale sensitivity to observation scale provides a means to distinguish between hierarchical competition and environmental filtering: Hierarchical competition consistently produces low trait diversity regardless of scale, whereas environmental filtering leads to a shift from trait similarity at fine scales to random patterns at broader scales.

### 3.4 Detectability of Assembly Processes

**FIGURE 4:**
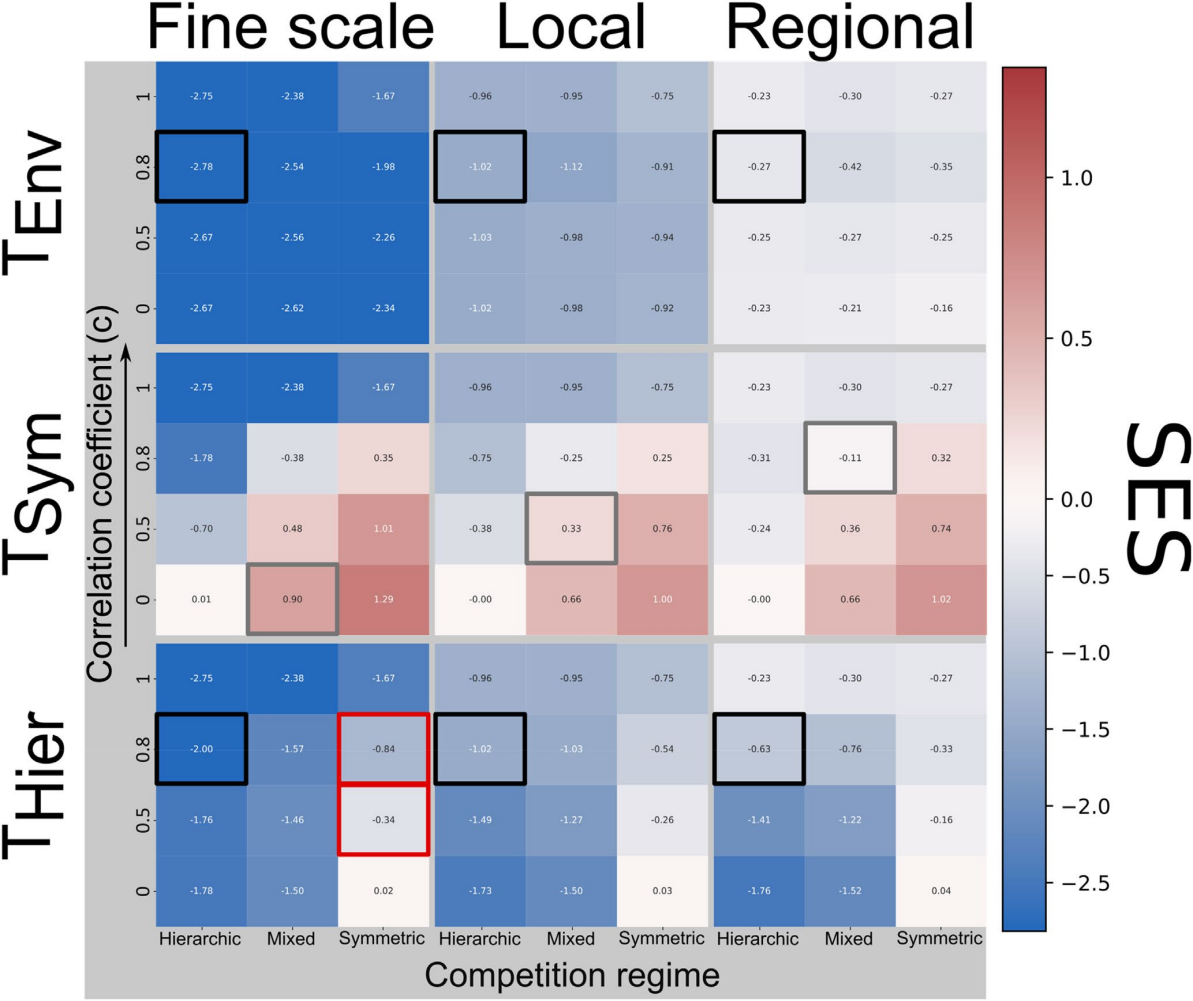
Heatmaps of the mean observed standardized effect sizes (SES) for the traits linked to environmental, symmetric and hierarchic filters (as rows) along the observation scales (as columns), the correlation between the traits and the underlying competitive regime (hierarchic, mixed, and symmetric). Cells are colored in function of the mean SES values of each specific case. Cells with a thick colored contour serve to highlight the specific cases discussed in the text.

Environmental filtering remained detectable at the fine scale across all competitive regimes and trait correlation levels. At the local scale, effect sizes were less pronounced but still indicated lower‐than‐expected trait diversity (mean SES = *−*0.96, 95% CI: [*−*0.97, *−*0.95]). At the regional scale, patterns were near‐random (mean SES = *−*0.26, 95% CI: [*−*0.27, *−*0.25]), with no evidence of high diversity of the environmental trait (Figure 4).

When considered independently, both symmetric and hierarchical competition produced clear and detectable patterns. In the absence of trait correlations, the signals of both competition regimes remained consistent across observation scales (Figures 2a and 3).

However, when both competition regimes jointly operated (i.e., mixed competition), detectability decreased, though signals remained discernible (Figure 3b). Under increasing trait correlation, the signature of symmetric competition weakened, shifting from trait dissimilarity to trait similarity (mean SES decreasing from 0.68 to *−*0.24, Figure 4, gray boxes).

The observation of a trait not involved in any assembly process, for example, the hierarchical trait, which was neutral in simulations involving only symmetric competition (Figure 2), provided limited information about assembly processes even when trait correlations were introduced. At low trait correlations (*c* ≤ 0.5), patterns of the neutral‐like trait diversity remained close to random (mean SES = *−*0.11, 95% CI: [*−*0.11, *−*0.10]). At higher trait correlations (*c* ≥ 0.8), patterns began to resemble those produced by environmental filtering (mean SES = *−*0.34, 95% CI: [*−*0.36, *−*0.32], Figure 4, red boxes).

Environmental filter and hierarchical competition both produced lower trait diversity than null expectations in their respective traits, making them indiscernible. However, at low trait correlations (*c ≤* 0.5), their responses to increases in observation scale diverged: Environmental filtering resulted in trait diversity shifting toward random assembly (mean SES increasing from *−*2.67 to *−*0.22), whereas hierarchical competition consistently maintained low trait diversity (mean SES from *−*1.79 to *−*1.76). At higher trait correlations (*c* ≥ 0.8), the distinction between the two weakened, as hierarchical competition became increasingly sensitive to observation scale (Figure 4, black boxes). This convergence may hinder differentiation between processes, particularly in empirical studies where detectability is influenced by factors such as sample size and species' environmental niche breadth (Supporting Information).

## 4 Discussion

Our study highlights the critical role of trait correlations and observation scale in shaping the detectability of community assembly processes from trait patterns. We identified three key findings:

- The combined effects of trait correlations and a too coarse observation scale can obscure the detection of symmetric competition.
- Symmetric and hierarchical competitions generate distinct trait diversity patterns, even when acting in concert. However, hierarchical competition and environmental filtering can produce similar trait patterns. Nevertheless, their different sensitivities to observation scale provide a means to distinguish them.
- The ecological filtering framework can successfully identify primary assembly processes even when multiple processes operate simultaneously, provided that the appropriate observation scale and relevant functional traits are chosen.

### 4.1 Identifying Assembly Processes From Trait Patterns

#### 4.1.1 Fine‐Scale Environmental Heterogeneity Limits the Detection of the Environmental Filter

Despite the combined effects of environmental filtering and symmetric competition on community assembly and the consideration of heterogeneous landscapes in the simulation model, trait patterns resulting from environmental filtering remained identifiable at both fine and local scales (see Figure 1C). This aligns with empirical findings demonstrating that fine‐scale environmental filtering can structure communities, even when competition operates at the same spatial scale (Conti et al. 2017; Pescador et al. 2020). Moreover, signatures of environmental filtering were detected not only at the fine scale but also at the local scale, where environmental conditions remain sufficiently homogeneous to select for similar species traits (de Bello et al. 2013; Monteiro et al. 2023). However, at the regional scale, where communities encompass a wide range of environmental conditions, environmental filtering signals were lost, resulting in trait diversity patterns resembling those expected under random assembly (Siefert 2012).

Contrary to some previous studies (Willis et al. 2010; Scherrer et al. 2019), we did not observe significant trait dissimilarity in environmental traits, even under high fine‐scale environmental heterogeneity. Trait dissimilarity emerged only under extreme environmental gradients or highly skewed distributions of species' environmental traits relative to the landscape (see Supporting Information). These conditions are rare in natural communities, suggesting that strong fine‐scale environmental heterogeneity is more likely to produce random trait patterns rather than dissimilar trait structures when communities are sampled at coarse observation scales.

#### 4.1.2 Environmental Dependence of Competitive Traits Limits the Detection of Competition

The effect of observation scale on trait patterns associated with symmetric competition was more limited than expected. Theoretical models suggest that signals of symmetric competition should be lost when communities are aggregated over multiple sites (Botta‐Dukát and Czúcz 2016), and empirical studies often report that competition‐driven trait dissimilarity is masked at large spatial scales due to the overriding effect of environmental filtering (de Bello et al. 2009; Siefert 2012; Chalmandrier et al. 2017; Mod et al. 2020; Bektaş et al. 2023). One explanation is that historical contingencies—particularly the identity of early colonizers—establish biotic contexts that constrain subsequent community assembly (Chase 2003). Early arrivals may preemptively occupy functional space, limiting the establishment of species with similar traits and resulting in locally dissimilar communities, but with different trait compositions across sites. When aggregated, these local trait dissimilarities produce random trait patterns at broader scales.

However, the consistent detectability of symmetric competition across scales was evident only in the absence of trait correlations. Since our interaction matrix is based on species trait differences, this generates a competitive regime that favors a specific set of trait values (Fort et al. 2009; Leimar et al. 2013). When the environmental and symmetric traits are uncorrelated, the trait values favored by competition remain the same across all cells, regardless of environmental conditions. In other words, there is an optimal combination of trait values that maximizes trait dissimilarity and minimizes competition. This suggests that the loss of competitive signals at larger scales in empirical studies may not only result from the spatial limitation of inter‐individual interactions but also from context‐dependent competition, where environmental filtering constrains the functional space available for competitive exclusion (Laughlin and Messier 2015).

An analogous and maybe more intuitive case was observed for hierarchical competition, which produced strong trait similarity at both small and regional observation scales. Using a spatially explicit model, Capitán et al. (2020) demonstrated that hierarchical competition can generate strong trait similarity across increasingly broad observation scales, even in the absence of environmental filtering. Similarly, hierarchical competition has been linked to consistent trait similarity across large areas, such as the dominance of similar maximum plant heights in herbaceous communities Capitán et al. (2021). The cases in our study where the traits are not correlated with each other correspond to a similar situation, where interspecies interactions are not conditioned by the environmental filter. Our simulations demonstrate that large‐scale detectable patterns can emerge from both hierarchic and symmetric competitive regimes, even when they both acted in concert to shape community assembly (Figure 3).

We suggest that the commonly detected loss of symmetric competition signals in trait patterns at larger scales (Götzenberger et al. 2012) may be due not only to the spatial limitation of interindividual interactions, but also to the context dependence of competitive outcomes due to interference from environmental filtering (Laughlin and Messier 2015). In other words, while a single trait combination maximizes trait dissimilarity, in the real world, the environmental dependence of the competitive traits leads to different trait combinations under different environmental conditions. In our study, this environmental dependence is expressed by the correlation between traits (i.e., the range of viable competitive traits is conditioned by local environmental conditions). Increasing trait correlation resulted in an overall decrease in the detectability of symmetric competition. Associated with the moderate scale effect, the signal of symmetric competition is lost at coarse observation scales (Figure 2). This is consistent with empirical evidence that competitive traits, such as plant height and specific leaf area, exhibit strong environmental dependence (Spasojevic and Suding 2012; Price et al. 2017; Poggiato et al. 2023), supporting the notion that environmental filtering shapes the functional landscape within which competition occurs (Laughlin and Messier 2015; Cadotte and Tucker 2017; Aguilar‐Trigueros et al. 2017).

#### 4.1.3 Hierarchical Competition Versus Environmental Filtering: The Importance of Scale Effects

Hierarchic competition consistently produced a high trait similarity pattern (*T*_Hier_) in our simulations. When traits were independent, this pattern remained stable across all observation scales (Figure 3), consistent with models and empirical studies showing that competitive dominance at the local scale can scale up to large‐scale spatial patterns (Gotelli et al. 2010; Capitán et al. 2020, 2021). In our study, this large‐scale trait pattern can be explained by a comparable underlying mechanism to the patterns from symmetric competition previously described. As in hierarchic competition, there is always an optimum trait value; the closer the species are to this value, the better they perform (Kunstler et al. 2012). Consequently, the outcome of competition can be the same over all the landscape when the hierarchical trait is not dependent on the environment, that is, non‐correlated traits. This is consistent with the scale effects observed in trait correlation (Figure 4).

However, hierarchical competition and environmental filtering generate similar trait patterns, making them difficult to distinguish in empirical studies (Mayfield and Levine 2010). Our results suggest that observation scale provides a key diagnostic tool:

- Environmental filtering: Strong trait similarity at fine scales, but a transition toward random patterns at broader scales.
- Hierarchical competition: Consistently strong trait similarity, regardless of scale, unless hierarchical traits are environmentally dependent (i.e., correlated with environmental traits).

This has important implications for trait‐based studies. Since a single trait can influence both environmental responses and species interactions (Lavorel and Garnier 2002; Price et al. 2017), explicitly considering trait–environment relationships is critical when interpreting trait similarity patterns.

#### 4.1.4 The Limitations of Multi‐Trait Diversity and Neutral Trait Correlations

Our study assessed whether multi‐trait diversity (i.e., aggregate functional diversity metrics) and neutral correlated traits could reliably differentiate assembly processes. Multi‐trait diversity primarily reflected the dominant process in terms of magnitude. Since environmental filtering was often stronger than competition, multi‐trait diversity frequently overemphasized the effect of environmental filtering, potentially masking competition. Neutral traits (i.e., traits not directly involved in assembly processes) exhibited random patterns at low correlation levels but mimicked environmental filtering at high correlation levels. This is consistent with previous theoretical predictions (Botta‐Dukát and Czúcz 2016). The tendency for environmental filtering to dominate trait‐based analyses may explain the overrepresentation of environmental filtering in empirical studies compared to competition (Götzenberger et al. 2012; Botta‐Dukát and Czúcz 2016).

### 4.2 Future Developments

The individual‐based simulation model used in this study captures the trait patterns resulting from environmental filtering, symmetric competition, and hierarchic competition under the explicit consideration of observation scales and trait correlations. However, several improvements could enhance this framework:

- Multidimensional trait response: We used a single trait to simulate the response of species to each assembly process. However, the characterization of species responses to environmental conditions and competition is thought to be a multidimensional issue (Mouillot et al. 2021). Incorporating multiple traits associated with each process could allow the exploration of responses to multiple environmental filters (Bello et al. 2013), for example, large‐scale macro‐climate versus small scale variation in soil depth. The case of competition is more complex, but it opens up a series of interesting questions about how to define species competitive interactions from species functional traits. A more mechanistic approach, like modeling explicitly resource limitation and individual uptake could help integrate environmental filtering and competitive interactions into a unified framework, linking explicitly competition to environmental gradients (Levine et al. 2025). In addition, deducing competitive interactions through resource use allows avoiding the hypothesis of a Gaussian shape for the competitive function (Falster et al. 2021).
- Mechanistic trait interactions: Here, we model the dependence between processes through the correlation of traits. However, we did not explicitly include the implication of traits in different demographic processes underlying community assembly. Including explicit links between traits and demographic processes would permit testing the utility of “soft traits” (loosely correlated with demographic parameters of multiple processes) against “hard traits” (e.g., demographic parameters of a single process such as maximum growth rate) (Lavorel and Garnier 2002).
- Competitive interactions in our model only take place at the finest scale (only between individuals sharing the same cells) and we focused our analyses on the variation of the observation scales. While it is commonly assumed that interactions mainly act at this small scale (McGill 2010), the exact spatial extent of species interactions remains unknown and is likely to vary between organisms (Zelnik et al. 2024). For instance, a tree will shade a larger area than a tall grass. The explicit modeling of interactions at the distance between individuals can be achieved by the addition of a Gaussian kernel to define the interaction range (Zelnik et al. 2024). Modeling interactions at varying distances between individuals could provide a more detailed understanding of how trait patterns scale up (Bektaş et al. 2023).

### 4.3 Recommendations for Empirical Applications

Our theoretical investigation of the identifiability of community assembly processes from trait patterns shows that when applied carefully and through the inclusion of relevant traits and observation scales, the filtering trait framework can be a successful framework to unravel assembly processes. We here draw a few recommendations for trait‐based empirical studies.

1. Optimize the sampling scale: Environmental structure and its heterogeneity strongly influence detectability. Measuring environmental variation and adjusting community sampling accordingly can improve detection of underlying processes (Viana and Chase 2019).
2. Prioritize ecologically meaningful traits: Traits that are mechanistically linked to assembly processes should be prioritized over correlated but functionally ambiguous traits (Lavorel and Garnier 2002; Violle et al. 2009).
3. Use independent traits when possible: Employing independent traits related to different processes enhances the ability to infer multiple concurrent assembly mechanisms (Rosbakh et al. 2022).
4. Account for scale effects: Hierarchical competition and environmental filtering produce similar patterns, but scale‐dependent changes in trait diversity provide a potential diagnostic tool to distinguish them. Aggregating communities from fine to broad scales may help separate these processes.

## Author Contributions

**Matthias Rohr:** conceptualization (lead), formal analysis (lead), writing – original draft (lead), writing – review and editing (equal). **Wilfried Thuiller:** conceptualization (supporting), supervision (equal), writing – review and editing (equal). **Loïc Chalmandrier:** writing – review and editing (equal). **Tamara Münkemüller:** conceptualization (supporting), supervision (equal), writing – review and editing (equal).

## Conflicts of Interest

The authors declare no conflicts of interest.

## Supporting information


Appendix S1.

## Data Availability

No data were used for this work. All the scripts needed to run the model and simulation experiments are accessible here: https://github.com/M‐Rohr/Trait‐patterns‐Model.
