# Supplementary material for: The Role of Observation Scales, Trait Correlations, and Competitive Regimes in Community Assembly Patterns
Source: Ecol Evol. 2025 Apr 24;15(4):e71272. doi: 10.1002/ece3.71272 (PMC12019707; doi:10.1002/ece3.71272)
Supplement: Supplementary file 1 — Appendix S1. [file ECE3-15-e71272-s001.docx]

**Supplementary materials** **for:**

**The role of observation scales, trait correlations and competitive regimes in community assembly**

**patterns**

# Sensitivity analysis of the fixed parameter

We conducted a sensitivity analysis to evaluate the impact of variations in our model’s fixed parameters (main text, Table 1). We tested a range of ±80% around the selected values, sampling 200 parameter combinations using a Latin hypercube design to uniformly explore the parameter space. For each combination, we ran 16 independent model realizations to account for stochasticity. We compared the resulting patterns, averaged over the 16 realizations and expressed as standardized effect sizes (*SES*), with those in the main text, measuring deviations as ∆*SES*.

This analysis assessed how each parameter influences trait patterns under mixed competition (*ϕ* = 0*.*5), both without (Figure S1) and with (Figure S2) trait correlation.

The sensitivity analysis indicates that the observed functional patterns are robust to large variations in most parameter values. Only the parameters directly linked to competition processes, specifically the competition niche breadth parameters ($\sigma_{S}$ and $\sigma_{H}$), affected the results. However, significant changes were only observed when these parameters varied by more than 30%.


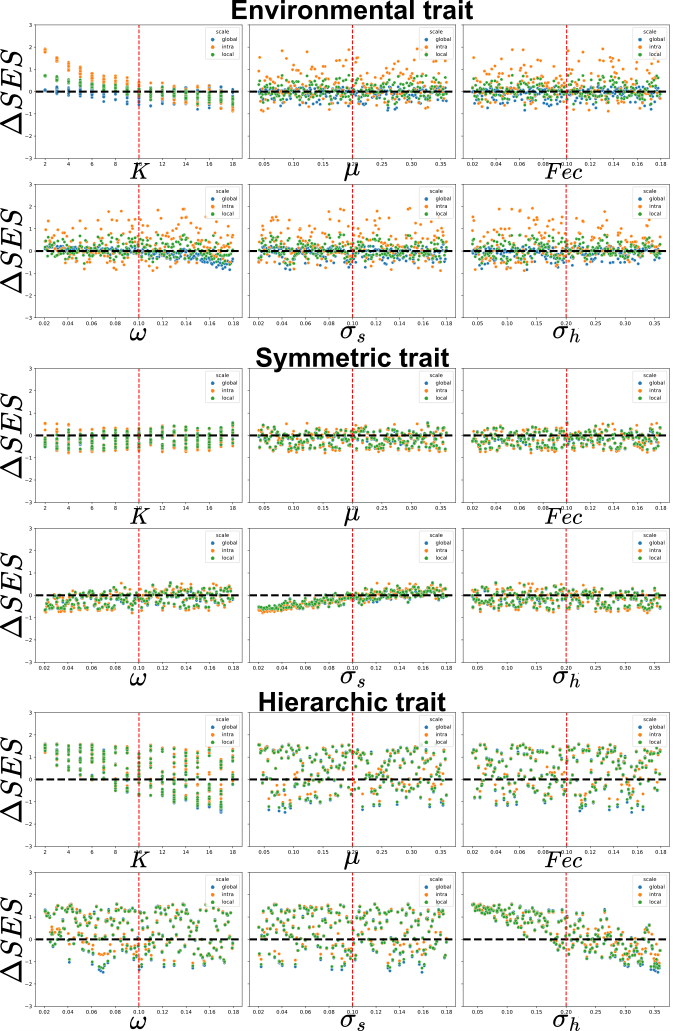


**Figure S1:** Sensitivity analysis of fixed parameters on standardized effect sizes (SES) of trait diversity. Each panel illustrates the effect of varying a fixed parameter on ∆*SES* for environmental, symmetric, and hierarchic traits across different spatial scales (global, local, and fine). ∆*SES* represents the deviation in standardized effect sizes of trait diversity compared to the baseline values used in the main text. Points represent the results of 200 simulations. The red dashed lines indicate the fixed parameter values used in the main text. No correlation among traits


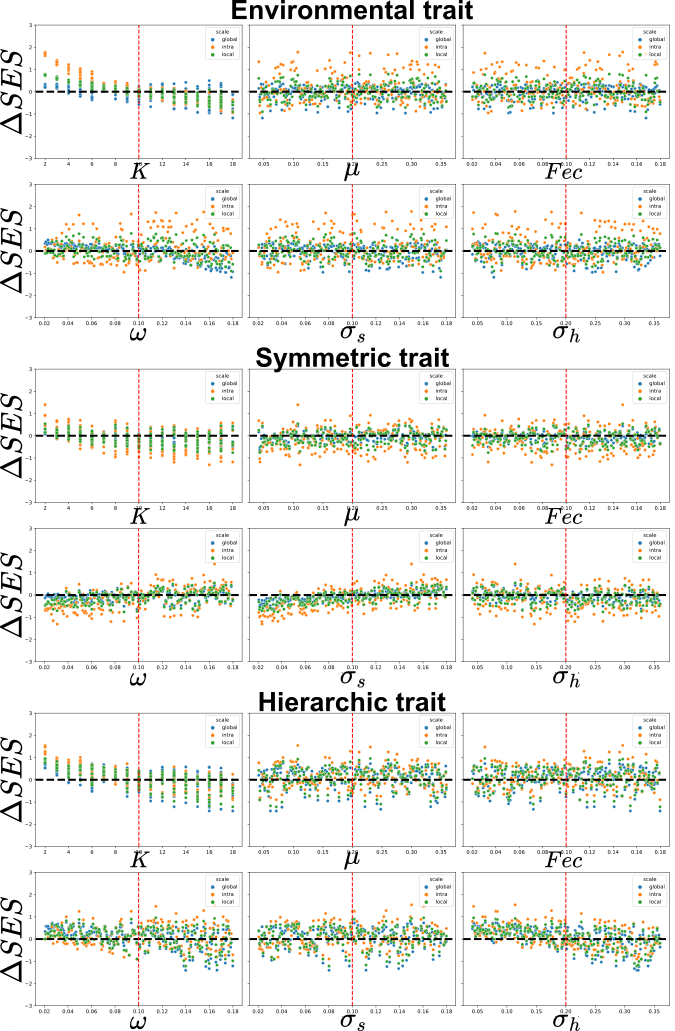


**Figure S2:** Sensitivity analysis of fixed parameters on standardized effect sizes (SES) of trait diversity. Each panel illustrates the effect of varying a fixed parameter on ∆*SES* for environmental, symmetric, and hierarchic traits across different spatial scales (global, local, and fine). ∆*SES* represents the deviation in standardized effect sizes of trait diversity compared to the baseline values used in the main text. Points represent the results of 200 simulations. The red dashed lines indicate the fixed parameter values used in the main text. High correlation among traits (correlation = 0.8)

# The model replicates expected patterns in standard situations


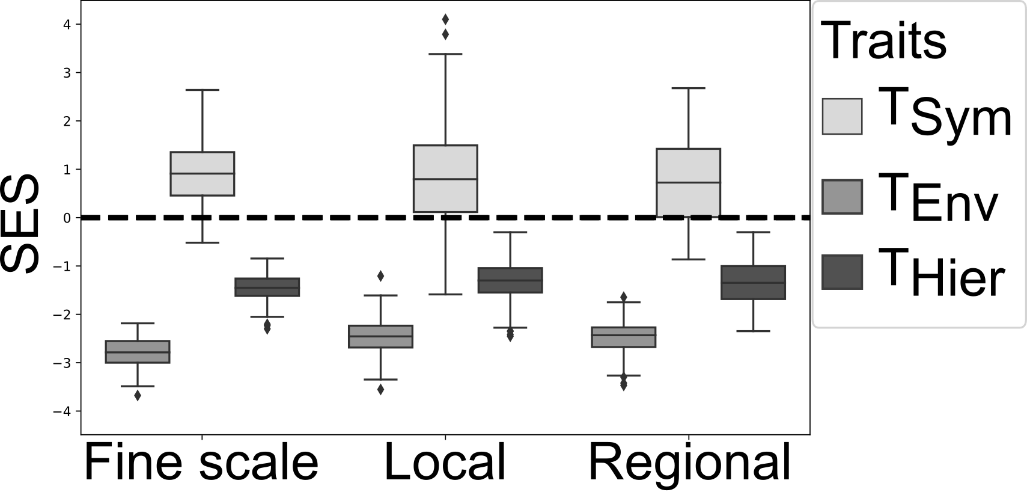
We first simulated communities in a simple standard case, *i.e.,* in a homogeneous landscape (*E_k_* = 0*.*5 in all cells) and without consideration of trait correlations, to verify that our assembly model produced the trait patterns expected from theory (Keddy, 1992; Cornwell et al., 2006) and earlier simulation studies (Münkemüller and Gallien, 2015) (Figure 3).

**Figure S3:** Standardized-effect-size (*SES*) of functional diversity for the three traits across different observation scales: Fine scale (cell’s communities), local scale (10*×*10 cell units), and regional scale (entire landscape). The communities were assembled in a homogeneous environment, the simulations considered environmental filter, symmetric competition and hierarchic competition as acting processes (*ϕ* =0*.*5).

We found that each of the three simulated processes produced the expected trait patterns compared to random assembly processes (Figure S3): Symmetric competition led to significantly higher diversity of the symmetric trait (*T_Sym_*), whereas both hierarchic competition and environmental filter led to significantly lower diversity of the hierarchic trait (*T_Hier_*) and the environmental trait, respectively.

# Species diversity of analyzed communities

To ensure that the simulated communities were diverse enough to analyse trait patterns comparable to highly diverse real-life communities (*e.g.,* alpine grassland), we calculated species diversity (as the Shannon index) at regional scale for different community assembly processes and different trait correlations.


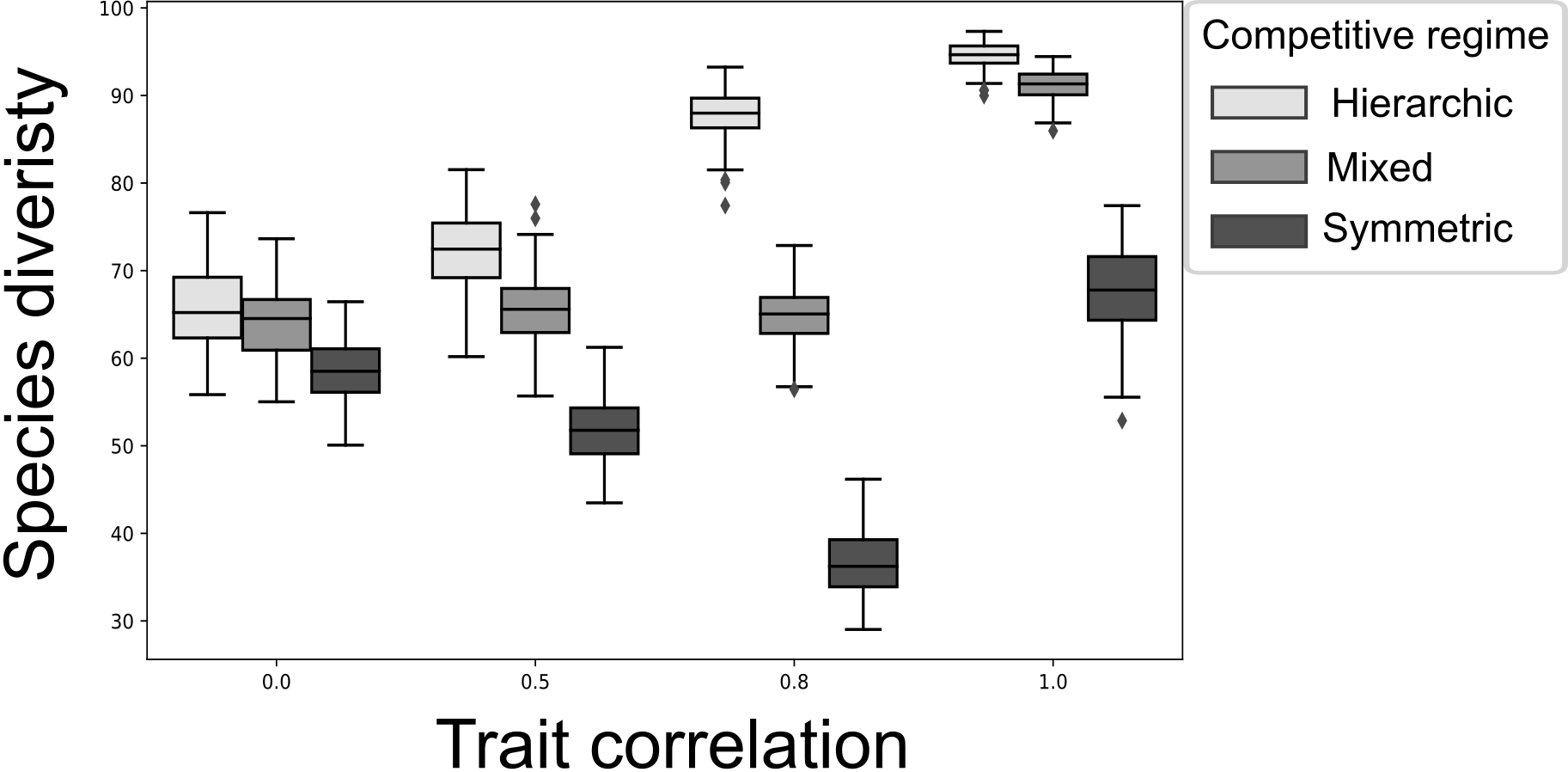


**Figure S4:** Species diversities (Shannon index) of simulation with environment filter and different competition regime in function of trait correlations. The species diversity was computed with the abundances at regional scale.

Regional scale diversity was high and comparable to realistic communities, with diversity index ranging from 29*.*0 to 97*.*3 (mean: 68*.*4 *±* 16*.*3).

Regional scale communities assembled through symmetric and hierarchical competition show different species diversity depending on the underlying assembly processes but all communities were relatively species rich. Symmetric competition led to less diverse communities than hierarchic competition. The effect of trait correlations on species diversities varied between the two competitive regimes, with a negative effect for symmetric competition and a positive effect for hierarchic competition (Figure S4).

This contrasting effect of competitive regimes on species diversity can be explained by two aspects of the model: First, intraspecific competition is high in both competitive regimes, preventing the dominance of a single species, even in the case of hierarchical competition. Second, as trait correlations increase, the effect of the environmental filter favouring species with similar competitive traits becomes more influential. This results in low-intensity competition in the case of hierarchical competition (the intensity of hierarchical competition is low between two species with similar traits). Conversely, in the case of symmetric competition, the favouring of similar traits increases competition and thus reduces coexistence.

# Multivariate trait patterns

In the main text trait diversity was computed independently for each trait. Here, we calculated multivariate trait diversity for all traits together because this is what is done in many applied

studies.


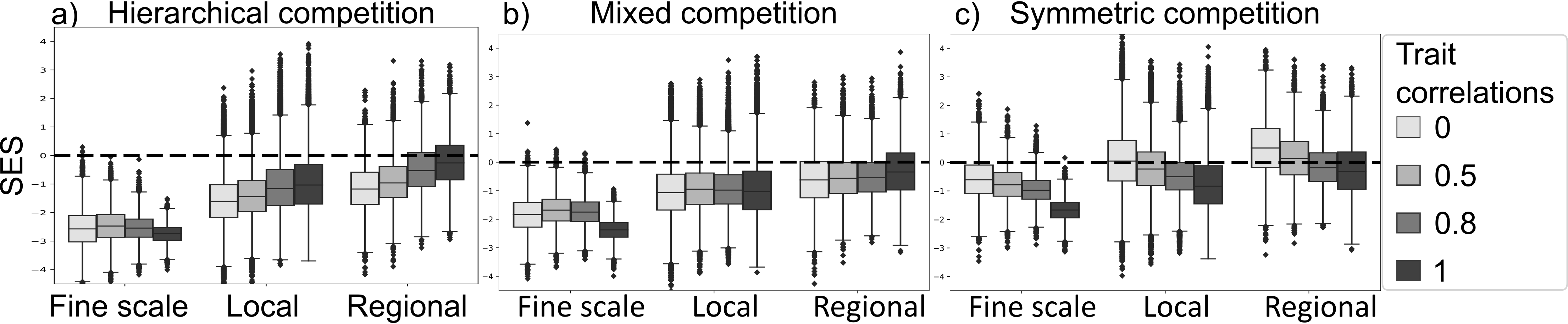
Under mixed competition, the summation of trait pattern in the multivariate trait diversity only permitted the identification of a pattern of low diverse community (Figure S5.b). High trait diversity related to symmetric competition was only detectable when the filter acted alone and not in combination with environmental filter and/or hierarchic competition. In other cases, the combination of opposing effects of the environmental filter (decreasing diversity) and symmetric competition (increasing diversity) blurred the patterns and the overall trait diversity was random (Figure S5.c).

**Figure S5:** Standardized effect size (*SES*) of functional diversity for the multivariate metric across different scales of observation: Fine scale (cell’s communities), Local scale (10*×*10 cell units), and Regional scale (entire landscape). The effects of various levels of trait correlations (from 0 to 1) are shown in three simulation experiments: a) Only hierarchic competition (*ϕ* =0), b) Mixed competition regime, hierarchic and symmetric competition (*ϕ* =0*.*5), c) Only hierarchic competition (*ϕ* =1). The communities are simulated in an auto-correlated environment.

# Effects of environmental heterogeneity and species niche breadth

To investigate more deeply the variety of trait patterns that be resulted from environmental filter, we tested the effect of both the environmental niche breadth ($\omega$) and spatial structure of environmental heterogeneity on the patterns of the environmental trait. We generated two different types of species pools, a "specialist" and a "generalist" pool which differ in their niche breadth ($\omega$), respectively equal to 0*.*1 and 0*.*5. For each pool we simulated community assembly with three types of heterogeneous environments:

- Gradient: A steep gradient with discrete intervals (ranging from 0 to 1, with steps of 0.1).
- Auto-correlated: The same structure as describe in the main text.
-
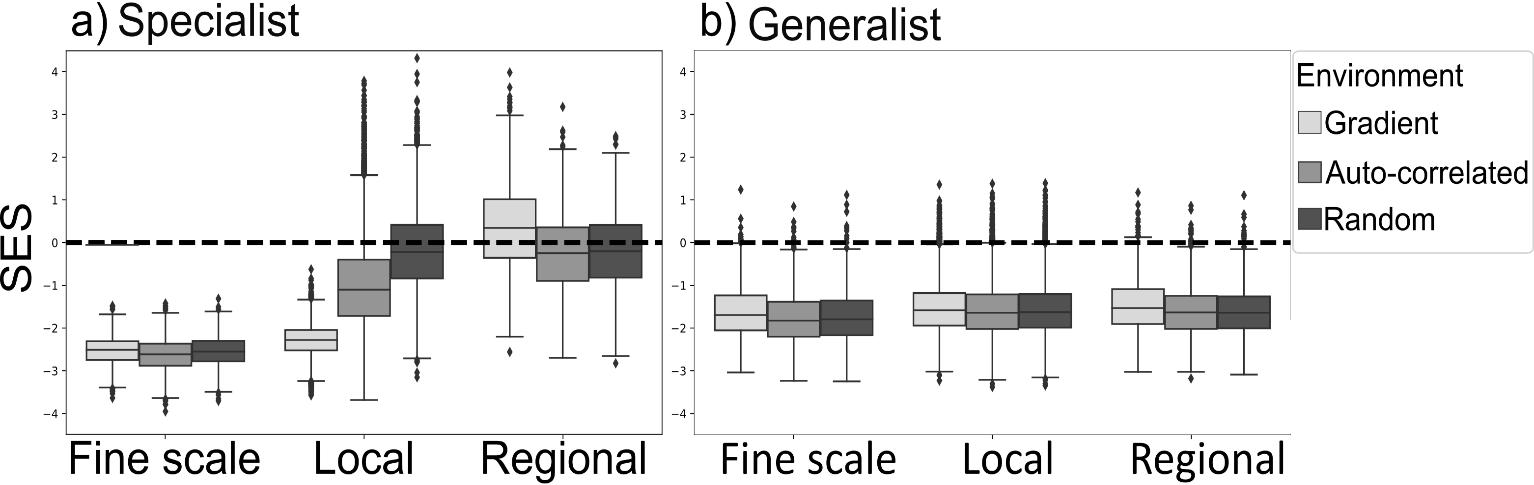
Random: Uniform distribution of the environmental values (*E_k_*) in space.

**Figure S6:** Standardized-effect-size (*SES*) of functional diversity for the environmental trait across different scales of observation: Fine scale (cell’s communities), local scale (10*×*10 cell units), and regional scale (entire landscape). a) The communities were assembled from a specialist species pool (niche breadth *ω* =0*.*1). b) The communities were assembled from a generalist species pool (niche breadth *ω* =0*.*5). For each communities three types of environmental structure were tested: Gradient, Gradient and Random.

For the specialist species pool (Figure S6.a), the response of the trait patterns depended on observational scale and environmental structure. For gradient and auto-correlated structures, the environmental conditions were similar at the local scale, resulting in low trait diversity. Conversely, with a random structure, the local scale was large enough to encompass all the diversity of environmental conditions, resulting in a random-like pattern. The specific case of the steep gradient resulted in high diversity at the regional scale. The large gradient with discrete interval sectioning and evenly spaced clusters of species, similar to symmetric competition, also exhibited high diversity.

For the generalist species pool (Figure S6.b), the observed communities showed low trait diversity across all observation scale. In this case, the environmental variation of the landscape was low in comparison to the size of the environmental niches of species, enabling species to perform well in every cell in the landscape. Yet, species with traits close to the mean of the environmental values had more cells in which they perform better than species on the edge of the environmental trait axis and are favoured in competition for space. This resulted in trait similarity at all observation scales.

# Effect of trait and environmental variable distributions from environmental filter-only patterns

We explored how different distributions of environmental values (*E_k_*) and values of environmental traits (*T_Env_*) affect trait patterns emerging from environmental filter. In particular we opposed the results obtain between an uniform distribution (as in the main text) and a normal distribution


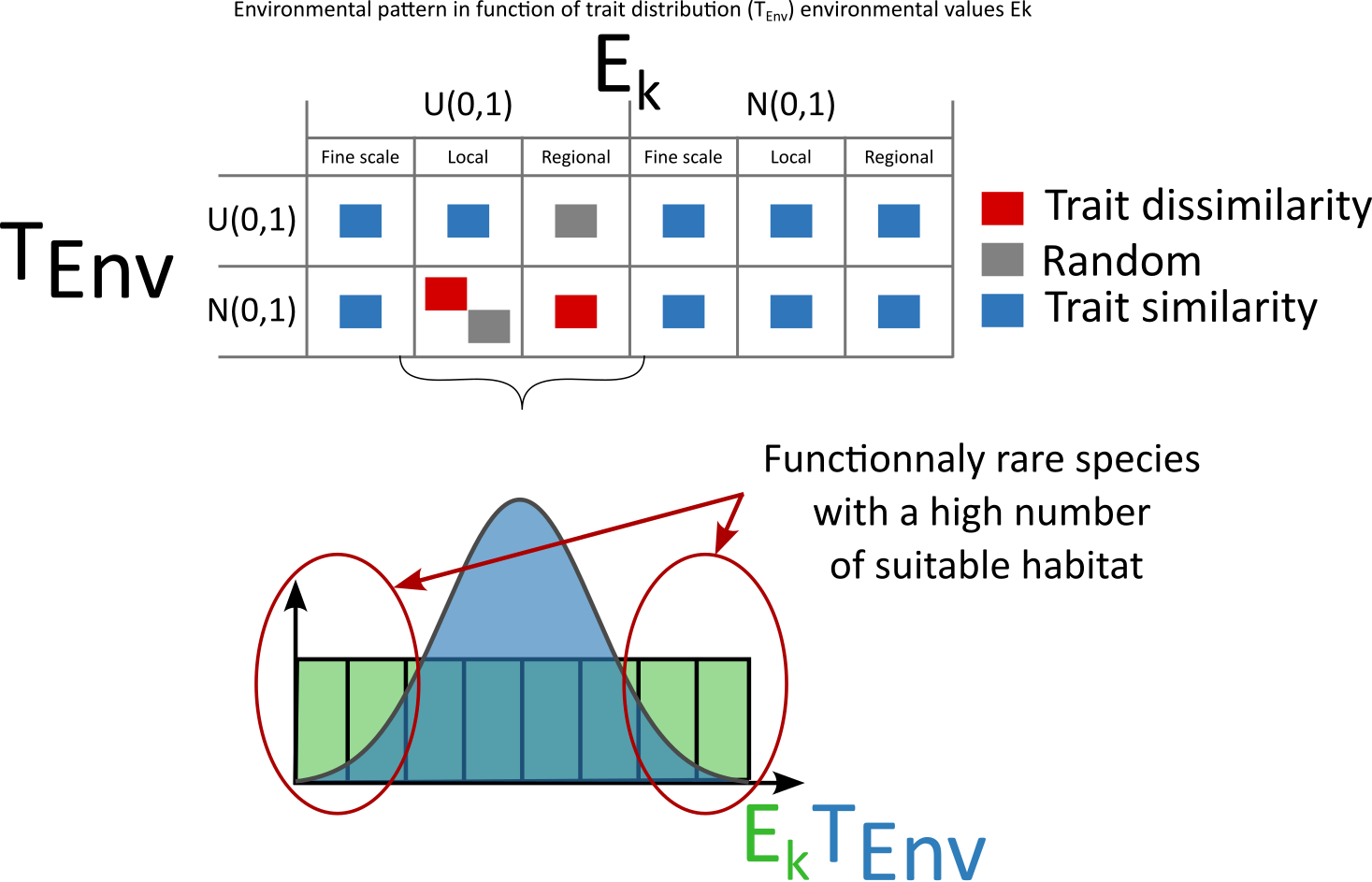
(*N*(0*,*1)). We also analysed the effect combining normally distributed trait values with uniformly distributed environmental values on the resulting patterns for the environmental trait (Figure S7).

**Figure S7:** Schematic representation of the trait patterns for the environmental trait for different distribution of the trait value (*T_Env_*) and the environmental values (*E_k_*). The scheme explain the cases where environmental filter results in trait dissimilarity.

Overall, we found that our results did not qualitatively change when switching the distribution assumption of our traits from uniform to normal under normally distributed environmental values. With this distribution, species that have their environmental trait (*i.e.*  their optimum) close to the mean value of *E_k_* benefit from a higher number of suitable cells, leading to a regional scale trait similarity, despite the environmental heterogeneity.

Only in the combination of uniformly distributed environmental values and normally distributed traits, we found that environmental filtering could result in greater trait diversity than random expectation. In this scenario, species situated at the border of the trait distribution (*i.e*. those that are functionally rare) are favoured by a high number of suitable habitats relative to the number of species. Conversely, species with trait values close to the mean of the distribution are subject to intense competition for space (Figure S.7). This disparity in habitat availability increases the population density of species situated at the border of the trait distribution at the regional scale, resulting in a community that appears more diverse than would be expected by chance.

# References

Cornwell, W. K., Schwilk, D. W., and Ackerly, D. D. (2006). A Trait-Based Test for Habitat Filtering: Convex Hull Volume. *Ecology*, 87(6):1465–1471.

Keddy, P. A. (1992). Assembly and response rules: two goals for predictive community ecology. *Journal of Vegetation Science*, 3(2):157–164.

Münkemüller, T. and Gallien, L. (2015). Virtualcom: A simulation model for eco-evolutionary community assembly and invasion. *Methods in Ecology and Evolution*, 6(6):735–743.
